# In Vitro Study of Calcium Microsecond Electroporation of Prostate Adenocarcinoma Cells

**DOI:** 10.3390/molecules25225406

**Published:** 2020-11-19

**Authors:** Aleksander Kiełbik, Wojciech Szlasa, Olga Michel, Anna Szewczyk, Mounir Tarek, Jolanta Saczko, Julita Kulbacka

**Affiliations:** 1 Faculty of Medicine, Wroclaw Medical University, 50-367 Wroclaw, Poland; aleksander.kielbik@outlook.com (A.K.); wojciech.szlasa@outlook.com (W.S.); 2 Department of Molecular and Cellular Biology, Wroclaw Medical University, 50-556 Wroclaw, Poland; olga.michel@umed.wroc.pl (O.M.); a.szewczyk@umed.wroc.pl (A.S.); jolanta.saczko@umed.wroc.pl (J.S.); 3 Department of Animal Developmental Biology, Institute of Experimental Biology, University of Wroclaw, 50-328 Wroclaw, Poland; 4 Université de Lorraine, CNRS, LPCT, F-54000 Nancy, France; mounir.tarek@univ-lorraine.fr

**Keywords:** electroporation, calcium, prostate cancer, focal therapy

## Abstract

Electroporation, applied as a non-thermal ablation method has proven to be effective for focal prostate treatment. In this study, we performed pre-clinical research, which aims at exploring the specific impact of this so-called calcium electroporation on prostate cancer. First, in an in-vitro study of DU 145 cell lines, microsecond electroporation (μsEP) parameters were optimized. We determined hence the voltage that provides both high permeability and viability of these prostate cancer cells. Subsequently, we compared the effect of μsEP on cells’ viability with and without calcium administration. For high-voltage pulses, the cell death’s mechanism was evaluated using flow-cytometry and confocal laser microscopy. For lower-voltage pulses, the influence of electroporation on prostate cancer cell mobility was studied using scratch assays. Additionally, we applied calcium-binding fluorescence dye (Fluo-8) to observe the calcium uptake dynamic with the fluorescence microscopy. Moreover, the molecular dynamics simulation visualized the process of calcium ions inflow during μsEP. According to our results calcium electroporation significantly decreases the cells viability by promoting apoptosis. Furthermore, our data shows that the application of pulsed electric fields disassembles the actin cytoskeleton and influences the prostate cancer cells’ mobility.

## 1. Introduction

Cell membrane integrity can be largely affected when the latter are exposed to pulsed electric fields (PEFs) of high enough intensity. The application of such electric pulses is believed to trigger formation of highly permeable spots (domains) in their lipid membrane [1,2]. This phenomenon is called electroporation (EP), and the changes induced depend on the intensity of the applied electric fields and can be either reversible, i.e., cells recover their integrity, or irreversible, in which case, the cells turn necrotic [3]. Electroporation has found use in the clinic as a non-thermal focal ablation method and has been applied as a minimal-invasive treatment of patients with internal organs tumors [4,5,6]. For focal prostate treatments, although several clinical trials showed promising results [7,8], IRE still remains at the investigation stage.

The reversible EP provides a broader spectrum of applications: It allows transfer of genes [9,10], drugs [11,12], small exogenous proteins, and other molecules [13,14]. It has been used, for instance, in clinic to increase the uptake of cytostatic drugs in a procedure known as electrochemotherapy [15]. Recently, we have described the technique of electroporation-based treatments in urology [16] and its development perspectives.

To avoid the potential harmful systemic effects of electroporation, and other drawbacks such as the difficult preparation and storage of cytostatic drugs, the use of calcium ions has been proposed as an alternative to traditional electrochemotherapy. This novel method termed calcium electroporation (CaEP) had been first presented by Frandsen et al. (2012) [17] and in no time proved its effectiveness in vivo and in vitro for treatments of different cancers [18,19,20]. Currently, clinical trials involve the application of calcium electroporation for skin malignancies (clinicaltrials.gov #NCT04259658), as a neoadjuvant therapy for early colorectal cancer (clinicaltrials.gov #NCT03694080), as a palliative treatment of head and neck tumors (clinicaltrials.gov #NCT03051269) as well as for advanced, inoperable colorectal cancer (clinicaltrials.gov #NCT03542214).

The present in vitro study aims to evaluate the effect of CaEP on prostate cancer model. Every year over one million patients are diagnosed with prostate cancer (PCa) [21]. The prevalence and mortality rate of PCa increases significantly with age. 55% of all deaths due to PCa concerns patients older than 65 years [22]. Nevertheless, the common risk connected to low and arguably to medium-risk PCa is an overtreatment [23]. The vast prevalence of PCa calls for effective, safe, and minimally-invasive therapy [24]. The fast development of new imaging methods such as multiparametric magnetic resonance imaging inevitably increases the meaning of focal therapies in PCa treatment [25]. Furthermore, recent researches show that access to care and treatment significantly influences the survival rate of patients with prostate cancer [26]. Calcium electroporation requires only readily available calcium chloride and an adequate electric generator with electrodes. Accordingly its simplicity and affordable cost are an undeniable potential for CaEP to become an effective accessible treatment among populations of developing countries [27].

Calcium EP has proved in in vitro and in vivo trials to have a selective effect on cancer cells [28]. To enhance the selectivity, it is required to minimize the occurrence of irreversible electroporation and boost rather the reversible one. Note that during CaEP, the death of reversibly electroporated cells results mainly from calcium influx [29], and cancer cells, with impaired mechanisms of calcium homeostasis, are those predominantly damaged [30,31].

Primary, in this study, the electrical voltage on electrode promoting the reversible electroporation has been determined.

Different cancer types might present different sensitivity to CaEP [32]. To establish whether CaEP has potential as the focal PCa treatment, the impact of CaEP on prostate adenocarcinoma viability was evaluated. The research on other cell lines suggested that the interval time between the drug administration and electroporation may influence the effect of the therapy [33]. As the outcome varies depending on the cell type, consequently, in this study, the optimal time of drug administration has been investigated.

After the unsuccessful treatment of PCa, disease progress often involves the development of metastasis [34]. In around 20% of patients, the recurrence of PCa after the focal treatment with IRE occurs [35]. To prevent the progression of cancer to the metastatic stage, the therapy should decrease the cancer cell motility [36].

Calcium EP may trigger cell death through different mechanisms. The most commonly-evaluated are apoptosis and necrosis. The apoptosis as programmed cell death prevents the development of excessive immune reaction, providing the high selectivity of focal ablation [37]. Conversely, necrosis stimulates immune response resulting in local inflammation. For higher voltage, affecting cell viability, the prostate cancer cell death mechanism after electroporation was evaluated.

Finally, the study of CaEP investigates the dynamics of the process. Firstly, the time dependence between PEFs delivery and calcium influx as well as the dynamic of calcium efflux was observed under the fluorescence microscopy using the calcium-selective Fluo-8 dye. Secondly, microsecond CaEP was analyzed with the in-silico molecular dynamic study. Both mentioned methods provide excellent insight and a better understanding of CaEP mechanism.

## 2. Results

### 2.1. Effects of PEFs on Cancer Cells Viability and Permeability

**Figure 1:**
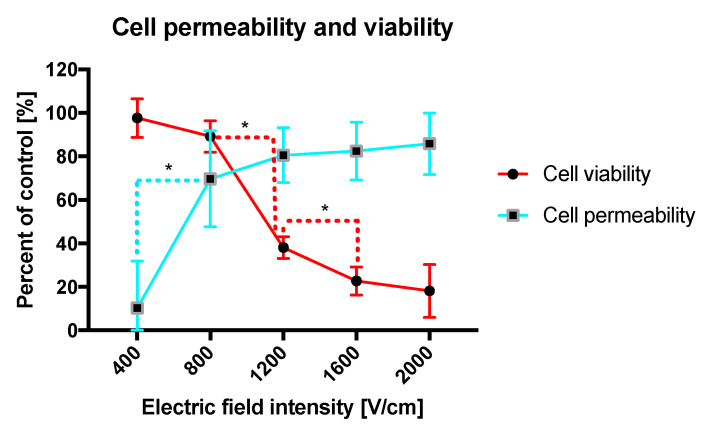
Viability and permeability of DU 145 cells as a function of electric field intensity. Suspended cells were electroporated in 4 mm cuvettes. The viability was assessed with MTT assay and permeability with YO-PRO^®^-1 uptake on the flow cytometry. Graphs are representative of 3 independent experiments. Data are mean ± SD (*n* = 3 independent experiments). (*) indicates statistically significant differences between the pair of samples electroporated with different electric field intensity (*t*-test, *p* < 0.05).

The impact of standalone microsecond short electric pulses was investigated on cancer cells suspended in the calcium free HEPES buffer. Figure 1 shows that the viability of cells decreases with the increasing intensity of the electric field. The most significant decrease occurs for electric fields intensities laying between 800 and 1200 V/cm. Note that even the highest electric field does not kill 100% of cancer cells. The curves indicate that permeability increases with higher electric fields intensities. The most significant increase in the permeability of the cells is detectable for fields between 400 and 800 V/cm. Above 1200 V/cm PEFs, almost all cells become permeable. Finally, the cells’ permeability does not appear to increase remarkably between 1200 and 2000 V/cm.

The results above enable the optimization of pulse parameters. The electric field intensity around 1000 V/cm provides the relatively high cell membrane permeability and does not decrease substantially the cell viability, indicating the highest ratio of reversibly electroporated cells. Once higher voltages on electrodes are applied, the cell permeability increases and viability decreases due probably to an irreversible electroporation of cancer cells.

### 2.2. The Influence of Time to Extracellular Calcium Application on CaEP Outcome

**Figure 2:**
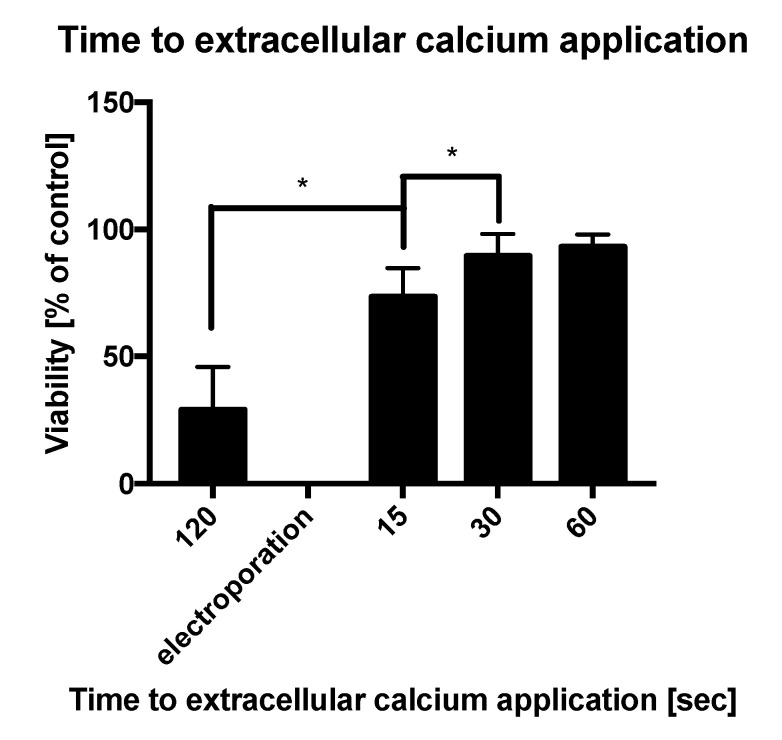
The influence of time to extracellular calcium application on DU 145 cells viability after exposure to pulsed electric fields (PEFs) (1000 V/cm). (*) indicates statistically significant differences between the pair of samples at a different time to calcium chloride administration (*t*-test, *p* < 0.05). Graphs are representative of 3 independent experiments. Data are mean ± SD (*n* = 3 independent experiments).

Figure 2 shows that the largest impact on the cells’ viability is achieved when calcium is added 2 min before the PEFs delivery. The administration of calcium after electroporation has a much lower influence on cell viability.

Overall, these initial data concerning the cells’ viability and permeability as well as the time to drug administration enable the optimization of the applied EP protocol for further investigation.

### 2.3. Effect of CaEP on Cancer Viability

**Figure 3:**
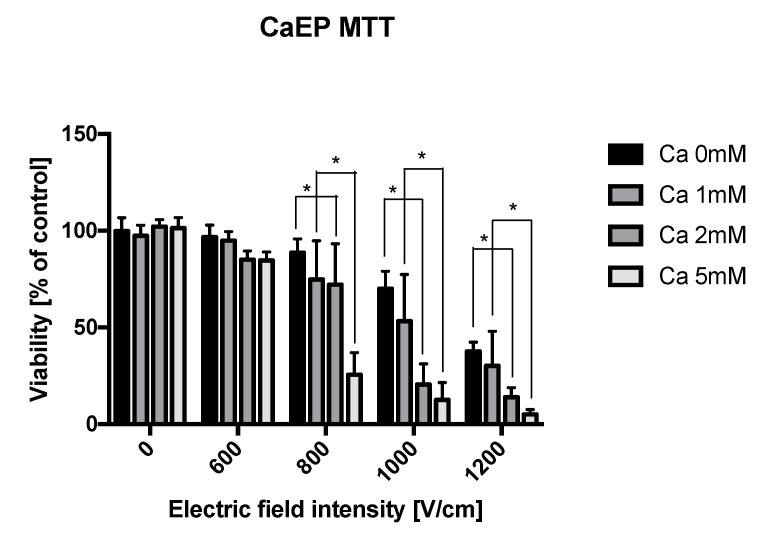
Viability of DU 145 cells after exposure to PEFs and calcium ions. The effect of EP was measured at three different calcium concentrations in HEPES buffer and HEPES buffer without calcium. Suspended cells were electroporated in 4 mm cuvettes. Graphs are representative of 3 independent experiments. Data are mean ± SD (*n* = 3 independent experiments). (*) indicates statistically significant differences between the samples at different calcium concentration (two-way analysis of variance (ANOVA) *p* < 0.05).

The viability of prostate cancer cells after CaEP was evaluated for different calcium concentrations namely 1 mM, 2 mM, and 5 mM and different pulse parameters (600 V/cm, 800 V/cm, 1000 V/cm, 1200 V/cm). Figure 3 shows viability of DU 145 cells after exposure to PEFs and calcium ions relative to control. Control represents the viability of not treated cells. The cytotoxic effect of the therapy increases with increasing electric field intensity. Higher calcium concentrations significantly lower the viability of the electroporated cells. This effect was not observed at the low electric field intensity of 600 V/cm. The synergistic effect of calcium seems to be most pronounced when 1000 V/cm PEFs follow the drug (Ca) administration. Finally, the standalone incubation with calcium ions without the application of PEFs does not change the viability of cancer cell.

### 2.4. Calcium Uptake Evaluation

**Figure 4:**
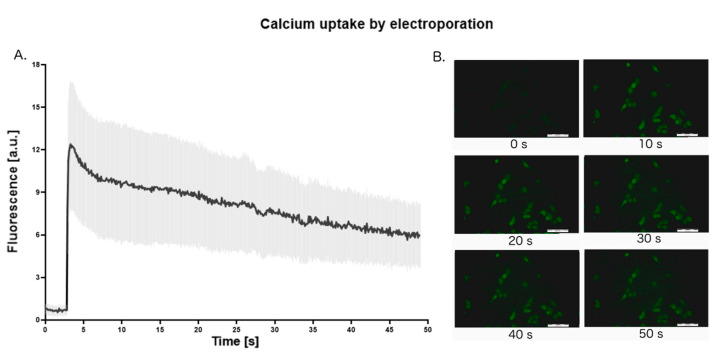
The dynamic of calcium uptake after microsecond electroporation (μsEP). The pulses were delivered after 3 s of initial observation. The whole observation period is 50 s. (**A**): The intensity of fluorescence as a function of time; (**B**): pictures of fluorescent cells taken every 10 s of observation. The graph represents the data form the three replicates of an individual experiment (*n* = 3 replicates).

To visualize the calcium uptake, we used the Fluo-8 dye. In cells, the latter is split by esterase to become fluorescent [38]. The intracellular calcium binds to the dye, increasing its fluorescence. The electric pulses of intensity ~800 V/cm were delivered after the onset of the data record. Figure 4 depicts the dynamic of the CaEP. During the delivery of the PEFs, the fluorescence starts to increase. The data indicates that the calcium uptake starts immediately after permeabilization. After reaching the optimum fluorescence, the cells begin to excrete calcium ions. At the beginning of reversion, the fluorescence undergoes an exponential decay and subsequently, the decay constant stabilizes.

### 2.5. Cell Death Quantification Assay after CaEP

**Figure 5:**
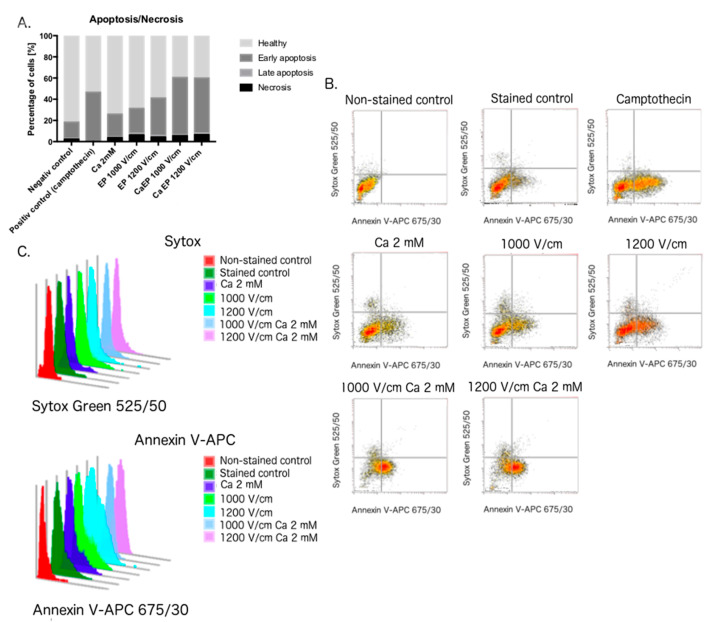
The distribution of apoptosis and necrosis in DU 145 cells after electroporation (EP) and CaEP measured by fluorescence of SYTOX™ Green and APC-Annexin V. The staining was performed 16 h after PEFs delivery. The graph represents the data form one individual experiment. (**A**): The distribution of healthy, early apoptotic, late apoptotic and necrotic cells after the treatment; (**B**): Flow cytometry dot plots. Each event is represented as a single point on a scatter-plot. Cells after 16 h incubation with camptothecin represent positive control for apoptosis. SYTOX™ Green fluorescence—forward scatter height; APC—Annexin V fluorescence—side scatter height; **C**: The overlay of histograms present fluorescence distribution for every sample.

To assess the mechanism of cell death following CaEP, we performed flow cytometry studies. 16 h after electroporation the cells were stained with Sytox Green dye and APC-Annexin V. The apoptosis was assessed by measuring the shift of phosphatidylserine to the outer leaflet of the cell membrane. Differently, late apoptotic and necrotic cells were stained with SYTOX™ Green—dye, which inflows only to the permeabilized cells and binds to cellular nucleic acids. Figure 5 shows that the CaEP affects tumor cells by triggering principally apoptosis. Calcium, when combined with PEFs, decreases further the viability of prostate cancer cells promoting hence apoptosis as well. The level of necrotic cells after the therapy does not differ among investigated samples.

### 2.6. Immunofluorescence Study of the Expression of Caspase-3

**Figure 6:**
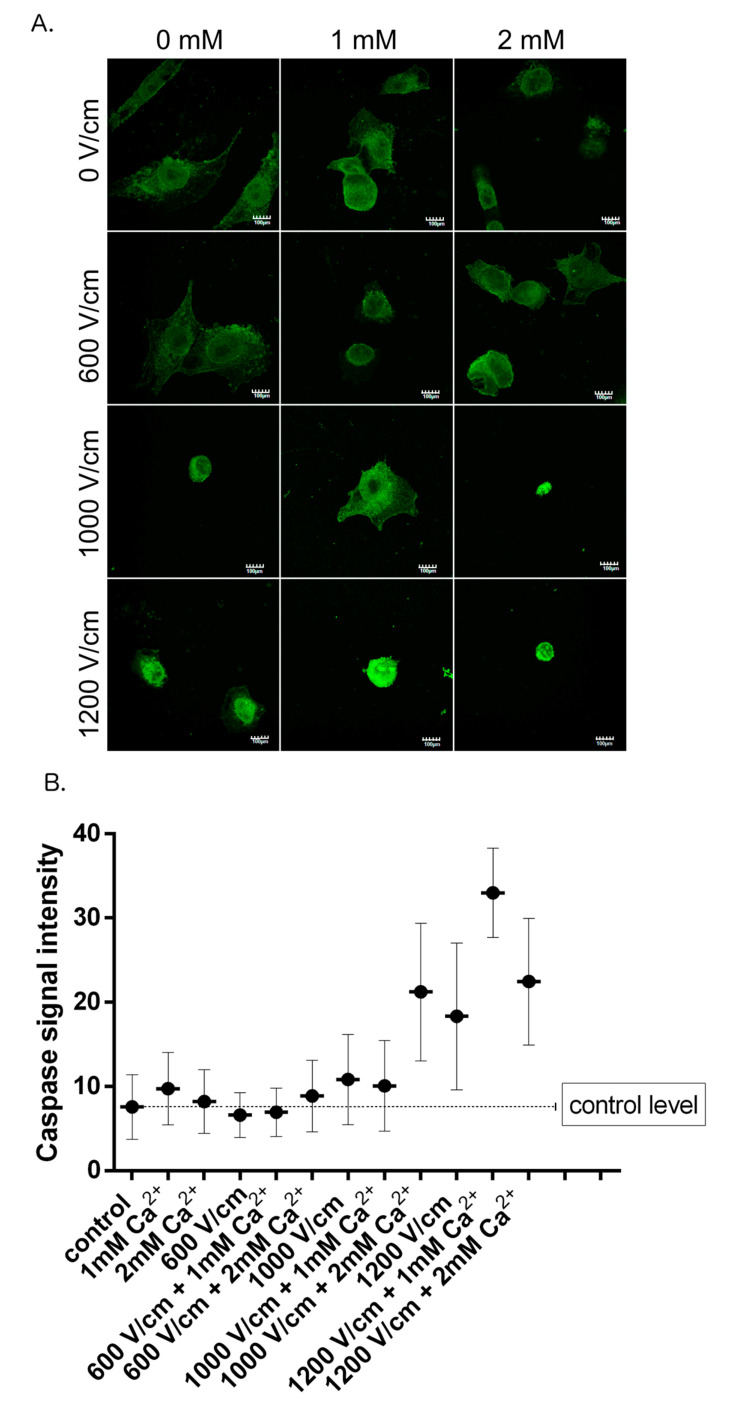
Confocal laser scanning microscopy (CLSM) visualization and intensity measurement of caspase 3 in DU 145 PCa cells after different therapy conditions. (**A**): CLSM pictures show the intensity of caspase-3 expression 16 h after the therapy; scale bar = 100 µm; (**B**): The caspase fluorescence signal intensity was analyzed by ImageJ software; The graph represents the data form the three replicates of an individual experiment. Data are mean ± SD (*n* = 3 replicates).

The immunofluorescence experiments were conducted to visualize the caspase-3 expression as key executor of apoptosis [39]. Caspase-3 can be activated via the intrinsic mitochondrial pathway (Bcl-2/Bax, Caspase-9), or via an extrinsic death receptor (Fas/FasL, Caspase-8) route [40]. Figure 6 shows that application of 600 V/cm PEFs with and without calcium does not change the expression of the caspase. In comparison, the fluorescence signal is remarkably stronger after the application of 1000 and 1200 V/cm PEFs (respectively 1.5-fold and 2.5-fold more intense compared to the control). Moreover, there is a difference in the expression between high-voltage PEFs with and without calcium. Namely, the 1000 and 1200 V/cm PEFs with 2 mM of calcium resulted in 2- and 1.25-fold higher fluorescence signal of caspase-3 (1000 V/cm + 2 mM vs. 1000 V/cm; 1200 + 2 mM vs. 1200 V/cm), respectively.

### 2.7. Effect of CaEP on Cancer cells Motility

**Figure 7:**
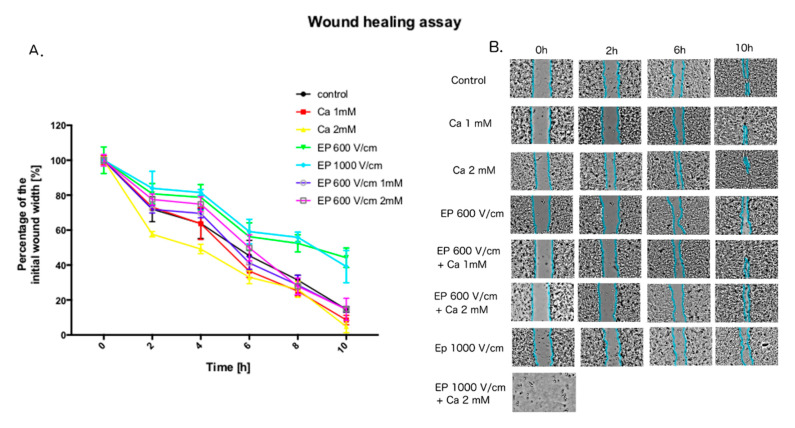
Effect of CaEP on cancer cell motility investigated with wound healing assay. The influence of different therapy protocols was investigated. (**A**): The percentage of a healed wound as a function of time. Images were analyzed by ImageJ software; (**B**): Images of wound gradually invaded by migrating cells. Images were taken in given time intervals. The graph represents the data form the three replicates of an individual experiment. Data are mean ± SD (*n* = 3 replicates).

The lower voltage PEFs affect the PCa cell viability significantly less, than the higher intensity electric fields. However, the application of low voltage PEFs has an effect on the mobility of cancer cells. Figure 7 reports the results of the wound healing assays. The cells were seeded inside the silicone inserts for 16 h before the latter were removed. The cells were scrutinized for 10 h until the separated colonies connected. The data shows that the application of PEFs impedes the mobility of cancer cell, while the effect has not been observed for lower-voltage CaEP. Noticeably, the effect of CaEP for higher intensity (1000 V/cm) PEFs could not be assessed as the protocol significantly influenced the cell’s viability, precluding the comparison of the created wound. The short time incubation with calcium does not show on the other hand any remarkable effect on the cell mobility.

### 2.8. Immunofluorescence Study of F-actin

**Figure 8:**
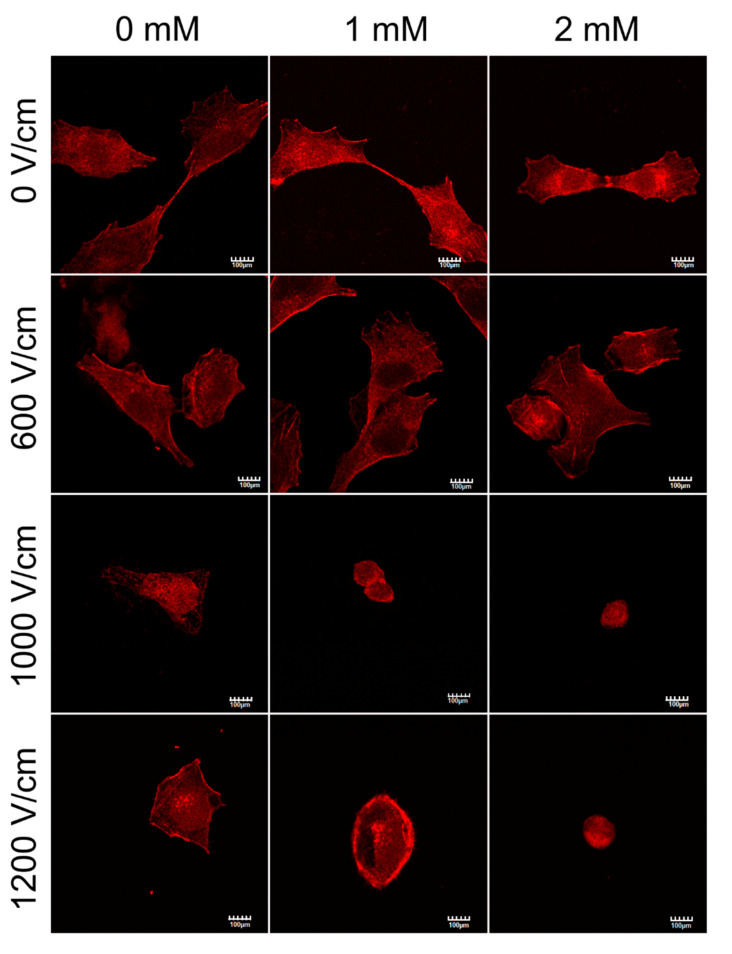
CLSM visualization F-actin structure in DU 145 prostate cancer (PCa) cells after different therapy conditions. Actin filaments were stained with fluorescein-conjugated phalloidin 16 h after the therapy; scale bar = 100 µm.

The cells were stained 16 h after the treatment with fluorescein-conjugated phalloidin. Figure 8 shows that non-treated DU 145 cells present a typical organization of F-actin fibers spanning the cytosol. Standalone incubation with calcium in low concentrations, does not influence the cytoskeletal structure of the PCa cells. Higher voltage PEFs increase the number of dying cells that have degranulated and fragmented actin filaments. 16 h after application of a 1000 V/cm PEFs, a loss of stress fibers and rounding of the cells occurs. When submitted to higher-voltage calcium electroporation, the cells create typical honeycomb-like structures due to actin accumulation on the cell periphery.

### 2.9. Molecular Dynamics Studies of CaEP

**Figure 9:**
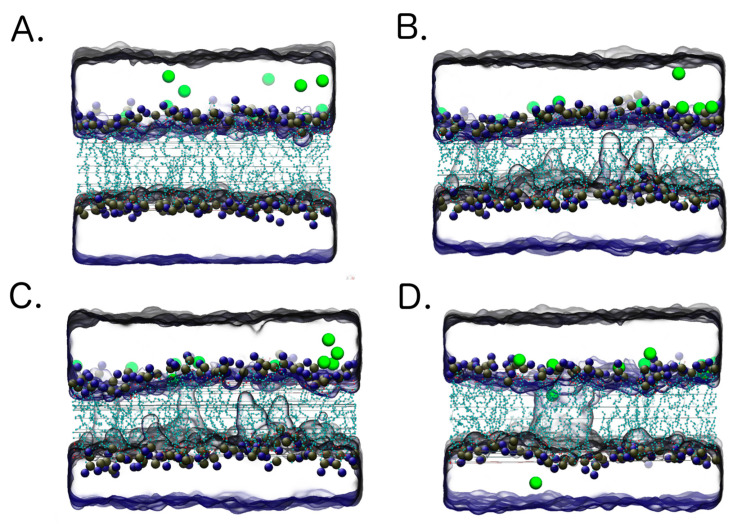
Molecular dynamics simulation of CaEP. Green molecules represent calcium ions. Numbers from (**A**–**D**) show the evolution of the process.

Molecular dynamic calculations were carried out to simulate the calcium ions distribution at the vicinity of the model lipid bilayer before and during electroporation. The microsecond electroporation was simulated by introducing the calcium ions imbalance (voltage) between the two compartments above and below the bilayer representing the extra and intra-cellular compartments. The electroporation process is visualized in Figure 9. Initially, calcium ions (green) show extramembrane localization (1). After an application of transmembrane voltage, the water molecules protrude to the membrane. Water fingers emerge on both sides of the lipid bilayer (2). Once, the water appendixes from both sides of the membrane contact, they form the water channel, which eventually spans the membrane. So, conducted electropores provide the possibility of calcium ions transport (3). With the increasing time of pore opening, more Ca^2+^ ions concentrate next to the pore opening, allowing more ions to cross the membrane (4).

## 3. Discussion

The intracellular calcium level is up to a thousand-fold lower than the extracellular one. To maintain this difference, the energy accumulated in ATP is required [41]. Inside the cell, calcium acts as a second messenger in biochemical processes and coordinates the changes in protein confirmations, so its level is required to be strictly controlled [41]. The equilibrium of cytoplasmic Ca^2+^ level is maintained in concentrations about 20 to 40 nM [42].

Electroporation can be used to increase the uptake of calcium and destabilize cancer cells. It was shown that CaEP leads to cancer death, decreasing cellular ATP level [29]. Moreover, it was recently shown that CaEP has an immunomodulatory effect. It converts a tumor microenvironment reducing the number of suppressor cells and increasing cytotoxic T-lymphocytes activity [43]. Besides, calcium electroporation affects tumor vasculature damaging blood vessels and potentiates as well the necrosis of tumor in vivo [44].

In our study, CaEP is shown to decrease the viability of prostate cancer cells triggering apoptosis. High intensity electric fields and calcium concentration enhance the therapy outcome. The apoptosis was assessed by measuring the expression of phosphatidylserine on the cellular surface that binds Annexin V and was confirmed with immunostaining of caspase-3. The mechanism of death induced by CaEP differs between the results published in the literature. Namely, some studies report apoptosis [45,46], others necrosis [17,44]. It is not that surprising, when considering the role of calcium in various paths of cell death’s mechanisms [47]. The main cause of death that triggered by standalone intracellular Ca^2+^ overload is generally apoptosis. Increased intracellular Ca^2+^ level activates chains of cell death effectors such as calcineurin, calpain, transglutaminase, endonucleases and phospholipases [48]. Moreover, Ca^2+^ overload results in permeabilization of the outer mitochondrial membrane, the release of mitochondrial proteins, and secondary activation of apoptotic events in other cellular compartments [49].

Necrosis due to calcium overload can also occur, however it would likely be a post-apoptotic necrosis where Ca^2+^ overload triggers caspase cleavage and inactivation of vital Ca^2+^-extruding proteins in the plasma membrane [48]. Recently, Gibot et al. (2020) have shown that the cell death after CaEP occurs as a result of mitochondrial dysfunction, without DNA damages, highlighting therefore the safety of such a therapy [50].

Importantly, our research shows that calcium, in order to strengthen the cytotoxic effect of the therapy, should be administrated before PEFs delivery. The effectiveness of CaEP on prostate DU 145 cell line is comparable to the one achieved on other cancer cell lines [17]. Most of the electroporation settings in the experiments followed the standard ESOPE protocol on EP, as it proved efficient and provides the possibility of outcome comparison between different trials [51]. Accordingly, only the applied voltage was adjusted to the initial results of cell permeability and viability after PEFs. In vivo studies showed that CaEP effect is more pronounced on an immunocompetent organism. The stimulation of the immune system results in an even complete tumor regression [52]. The next set of trials should investigate the in vivo effect of CaEP on prostate cancer.

Two classes of Ca^2+^ ATPases provide the stability of Ca^2+^ influx and efflux. In PCa cells the plasma membrane Ca^2+^ ATPase (PMCA) enables the transport of Ca^2+^ across the plasma membrane and SERCA transfer calcium within intracellular pools, as the one located in the endoplasmic reticulum [42]. The other mechanisms described of PCa Ca^2+^ clearance are the mitochondrial uniporter (MCU) and with a more limited scope the sodium-calcium exchanger (NCX) [53]. Moreover, the transient receptor potential superfamily (TRP) of cation channels and store-operated Ca^2+^-permeable channels (SOC) contribute to Ca^2+^ homeostasis of PCa [54].

A different response to CaEP was observed for healthy cells compared to cancer cells [46,55]. Frandsen et al. (2017) suggested that the rationale behind this is the lower expression of PMCA among cancer cells. Reduced level of PMCA might impede the efflux of calcium ions which results in higher Ca^2+^ concentration in cancer cells after electroporation [30]. Considering prostate cancer, with the progression of PCa to androgen-independent state, the changes of Ca^2+^ homeostasis such us SERCA downregulation and increased Ca^2+^ leak from endoplasmic reticulum occurs [56]. Moreover, the down-regulation of MCU in PCa has been observed [57]. Therefore, prostate cancer cells might have the restricted potential to store intracellular Ca^2+^ in organelles, and consequently exhibit a high susceptibility to CaEP. Undoubtedly, further studies are needed to confirm this hypothesis.

In our study, electroporation with low voltage pulses decreases the mobility of prostate cancer cells in vitro. Surprisingly, the effect was not as prominent once cells are electroporated in presence of calcium at low concentration. However, an extracellular concentration of calcium up to 2 mM is more physiological than no calcium at all. Once low voltage PEFs are delivered, cells electroporated in medium with physiological level of calcium preserve their mobility properties.

The study investigates whether the cell mobility changes observed after EP can be partially due to actin filaments disruption. Actin filaments play an important role in cancer progression and mobility [58]. Calcium is a well-known modulator of the cytoskeleton of the cell plasma membranes. In this study, we have shown that an increased intracellular concentration of calcium enhances the EP-induced actin disruption after higher voltage PEFs. We did not observe any correlation between actin disruption and impeded mobility after an application of PEFs at lower voltage. Several studies investigated the impact of electroporation (with and without calcium) on actin filaments. However, the wide spectrum of possible alterations in cytoskeleton triggered by PEFs application indicates that the effects strongly depend on the cell type [59].

Our study proves that after the application of PEFs prostate cancer cells deal with calcium overload either by extrusion or reuptake into intracellular organelles. After a few seconds, the decay constant stabilizes and cells maintain the high level of intracellular calcium. The possible explanation of this phenomenon might be the beginning of the cells apoptosis, which results in higher concentration of intracellular calcium [60].

Research evaluating the calcium uptake confirmed that Ca^2+^ peaks can be achieved with single pulse delivery [61]. Moreover, even in a calcium-free medium, the peak can be observed. It was demonstrated that a single μs PEF increases the intracellular calcium level acting on the endoplasmic reticulum [61]. Low-voltage CaEP, which does not affect cell viability, enables the investigation of Ca^2+^ level shifts and their impact on prostate cancer cells’ physiology. In this cited study, the fluorescence intensity curve shape is similar to the one obtained in our experiment.

Used in our experimentFluo-8 dye might not be the optimal dye for measuring the calcium uptake after the electroporation. However, this dye indicates the changes in intracellular Ca^2+^, which can occur either as a result of the inflow of extracellular Ca^2+^ or due to the release of Ca^2+^ from intracellular stores. The latter was not estimated in our study.

The live-cell calcium uptake experiments were performed on adherent cells. The adherent cells have a robust cytoskeleton, with cell-to-cell junctions. The cytoskeleton stabilizes the cell membrane and affects electroporation [62]. Moreover, the difference in shape between adherent cells and cells in suspension indicate that their transmembrane voltage in the same external electric field can differ [63]. Therefore, the calcium uptake curve of suspended cells might have a different shape. Accordingly, the results obtained from the calcium uptake study cannot support and explain the results and conclusion of the experiments performed on suspended cells.

Molecular dynamics simulations help model the phenomena through the numerical integration of multiple particle motion equations. In the case of CaEP it provides a mean of following at the molecular level the calcium uptake and the specifics of the calcium ions interactions with the lipid membrane [64]. Our model proves high affinity of the calcium ions to the bilayer, which results in the accumulation of the calcium beside the cells. We present that the process of calcium ions’ transport through the membrane is sequential and not all of the ions penetrate the membrane at once, even though the ionic gradient is present. Moreover, the simulation shows that electroporation does not result in immediately alignment of intracellular and extracellular calcium. MD simulations have already proved that the presence of Ca^2+^ has almost no effect on the pore lifetime [65]. Calcium changes though the conductivity of the electroporation buffer and consequently might have an impact on transmembrane voltage generation [66]. This effect is more pronounced once nanosecond PEFs are applied.

Given the promising in vitro results, a series of clinical studies investigating the outcome of CaEP in vivo have been conducted. In the majority of such studies, a higher calcium concentration of 168 mM calcium was injected into tumor tissues [27]. The volume of injected calcium chloride solution was ~50% of the volume of the tumor [27]. First a CaEP double-blind Phase 2 study on patients with cutaneous metastases proved the effectiveness and safety of the treatment. The Ca^2+^ solution was injected intratumorally, and the EPs were delivered immediately after the calcium administration. No serious adverse events were observed. The outcome of the therapy on cutaneous metastases was similar to that of electrochemotherapy using bleomycin [18]. One case report describes the systematic immune response after the CaEP treatment [67]. In another Phase I study the effect of CaEP was studied on patients with recurrent head and neck cancer [19]. Following CaEP, no serious adverse events were reported. No signs of hypercalcemia, or cardiac arrhythmias were observed after the Ca^2+^ intratumoral injections. Clinical responses were observed in three out of six patients. Moreover, one patient remained without any clinical evidence of cancer during 12 months of observation. Calcium was also applied with IRE for internal organs tumor treatment [68]. Although restricted, the present clinical experience with CaEP did not show any serious side effect of the therapy, thus the CaEP can be further investigated in clinical trials also for broader spectrum of tumors.

The present experience with electroporation-based technologies for PCa treatment concerns predominantly IRE [16]. One case report describes the application of electrochemotherapy with satisfying results [69]. For PCa treatment, the electroporation is applied for focal cancer ablation or in case of spread tumor for the whole gland ablation. Potential candidates for the therapy are patients with intermediate-risk PCa [16]. The electrodes are inserted transperineally under the guidance of ultrasound [35]. During IRE protocols patients require general anesthesia and muscle relaxation [70]. Although effective, IRE not always provides the total oncological control of the tumor [8]. Considering promising results in vitro, EP with calcium has a chance to enhance the oncological outcome of the therapy.

## 4. Materials and Methods

### 4.1. Cell Cultures

The human prostate cancer cell line DU 145 was obtained from the American Type Culture Collection. Cells were grown in monolayer cultures in Eagle’s Minimum Essential Medium, (EMEM, Sigma-Aldrich, Merck, Poznan, Poland) supplemented with 10% fetal bovine serum (FBS, Sigma-Aldrich) and antibiotics (penicillin/streptomycin; Sigma-Aldrich). Cells were kept under standard culture conditions at 37 °C in a humidified atmosphere containing 5% CO_2_. The cells were regularly rinsed with PBS and harvested with 0.025% trypsin solution (EDTA; Sigma-Aldrich).

### 4.2. Preparation of the Calcium Solutions

The later were prepared from the stock solution of calcium (CaCl_2_, Sigma-Aldrich dissolved in PBS (Sigma-Aldrich) at a concentration of 100 mM. Subsequently the required concentrations were achieved with dilution of stock in EP buffer to concentrations: 0 mM, 0.5 mM, 1 mM, 2 mM, 5 mM, and 10 mM.

### 4.3. Electroporation of Cells

The cells in the concentration of 5 × 10^5^ cells/mL were suspended in 300 μL HEPES buffer (10 mM HEPES (Lonza), 250 mM sucrose, and 1 mM MgCl_2_ in sterile water) with different concentration CaCl_2_ or in calcium free HEPES for controls. The cells were placed in a 4 mm cuvettes (BTX, Syngen Biotech, Poland). The square wave electroporator was used to deliver the electric pulses (ECM830 Square Wave Electroporation System; BTX, Syngen Biotech, Wroclaw, Poland). The cells were exposed to 8 pulses of 100 µs, 1 Hz, and 400, 800, 1200, 1600, or 2000 V/cm. Subsequently, the cells were incubated for 20 min and then suspended in the culture medium and placed in 96 or 6 well plates. All measurements were performed after a 24 h incubation.

### 4.4. Cell Viability Assay

To determine the cell viability, the activity of mitochondrial dehydrogenases was measured with MTT assays. First, the electroporation of suspended cells was performed following the protocol described above. Subsequently, after a 24 h incubation, the culture medium from 96 well plate was removed, and 100 μL of 0.5 mg/mL MTT (3-(4,5-dimethylthiazol-2-yl)-2,5-diphenyltetrazolium bromide, Sigma) in PBS buffer was added. After 3 h of incubation at 37 °C, a 100 μL acidified isopropanol (0.04 M HCl in the absolute isopropanol) was added to dissolve formazan crystals. Finally, the multiplate reader (GlowMax, Promega, Walldorf, Germany) was used to measure the absorbance at 570 nm. The results were expressed as the percentage of viable cells relative to untreated (control) cells.

### 4.5. Evaluation of the Influence of Time to Drug Administration on CaEP Outcome

Calcium chloride was added to the cell suspensions in cuvettes in different time intervals relative to electroporation. Each time, the final concentration of 2 mM calcium in buffer was achieved. To electroporate the cells, 1000 V/cm pulses were delivered according to the protocol described above. The cell viability was measured according to the viability assay.

### 4.6. Cell Permeability Quantification Assay

The cells were electroporated according to the protocol described above. Prior to electroporation, the green-fluorescent dye YO-PRO^®^-1 stain (Y3603, Thermo Fisher Scientific Inc., Warsaw, Poland) in the concentration of 100 μL/L was added to buffer. YO-PRO^®^-1 cellular uptake reflects the degree of the plasma membrane permeabilization [71]. The EP cells were then centrifuged and diluted in 0.5 mL PBS. The green Fluorescent intensities were detected on the Cube 6 flow cytometer. YO-PRO was excited with a 488 nm laser and measured with the FL-1- detector (525/50). The results are expressed as the percentage of permeabilized cells.

### 4.7. Cell Death Quantification Assay

The cell death mechanism was detected with flow cytometry. The cells were electroporated according to the description above and left incubating in the culture medium for 24 h. In the next step, the cells were harvested with trypsin and centrifuged. Subsequently, the cells were resuspended in 0.5 mL PBS containing SYTOX™ Green Nucleic Acid Stain (Thermo Fisher Scientific, Warsaw, Poland) and APC bounded Annexin V from the APC Annexin V Apoptosis Detection Kit (BioLegend, San Diego, CA, USA) as described in BioLegend instructions. Flow cytometry was performed with a Cube 6 flow cytometer. The fluorescence of Sytox was excited with a 488 nm laser and measured with the FL-1 detector (525/50) and APC fluorescence was excited with 640 nm laser and measured with the FL-4 detector (675/30).

### 4.8. Calcium Uptake Evaluation

A volume of 50 μL of cell suspension in the concentration of 10^5^ cells/mL was placed on the microscope slide. After 24 h the medium was removed, cells were stained with 4 µM Fluo-8 diluted in PBS and left incubating for 20 min. The slide was placed on the microscope stage with the electrode touching its surface. Cells visible in the objective were directly placed between two needles of the electrode BTX533 (BTX, Syngen Biotech, Poland). The PBS on the microscope slide was replaced with electroporation buffer HEPES with calcium at a 2 mM concentration. Subsequently, the cells were electroporated according to the protocol with field intensity reaching around 1000 V/cm. The increase in cell fluorescence during the electroporation was observed with the fluorescent microscope Olympus BX53F2 (Olympus, Tokyo, Japan). The changes in cell fluorescence were evaluated with ImageJ [72].

### 4.9. Immunofluorescence Studies of Actin Cytoskeleton and Caspase-3 Expression

The cells were electroporated according to the protocol described above. Subsequently the cells were seeded on glass coverslips places in 6 well plate and incubated for 16 h at 37 °C and 5% CO_2_. then the samples were washed with PBS (BioShop, dist. Lab Empire, Rzeszow, Poland), fixed in 4% formaldehyde (Roth, Germany) and washed with PBS again.

For the caspase-3 expression assessment, following fixation, the cells were incubated for 5 min with PBS with 1% Triton 100X, washed with PBS and left for 60 min with 1% HS, and the primary anti-caspase 3 antibody (1:100, sc-7272, Abcam, UK), diluted in PBS and incubated for 60 min at 37 °C and 5% CO_2_. Subsequently, the samples were washed 3 times with PBS, and incubated with Alexa Fluor 488 secondary anti-mouse antibody (Ex. 490 nm, Em. 525 nm; 2 μg/mL, A11029, Invitrogen) for 60 min at 37 °C and 5% CO_2_. After the washing in PBS, the cells were mounted with DAPI Mounting Medium (Roth, Germany).

For the actin cytoskeleton staining, the cells were incubated for 5 min with PBS with 1% Triton 100X, washed in PBS and left for 60 min with 1% HS, and the Alexa Fluor™ 594 Phalloidin (Ex. 581 nm, Em. 609 nm; 2 μg/mL, A22283, Life Sciences—Thermo Fisher Scientific), diluted in PBS and incubated for 60 min at 37 °C and 5% CO_2_. After washing in PBS, the cells were mounted with DAPI Mounting Medium (Roth, Germany).

The samples were analyzed with a confocal laser scanning microscope with using laser wavelengths: 405 nm, 473 nm and 559 nm; 60X oil immersion objective lens with 1.35 NA (Olympus FluoViewer 1000, Tokyo, Japan).

### 4.10. Wound Healing Assay

To investigate the effect of CaEP on the migratory capacity of cells, the wound healing assay was performed. The cells were electroporated following the protocol described above. After EP, the cells were centrifugated and diluted in the culture medium. The silicone insert was applied in to form a 500-μm ± 50 μm cell-free space between colonies. Cells were incubated at 5% CO_2_ and 37 °C for 16 h to stick to the bottom and create the monolayer. Subsequently, the silicone insert was removed. Images of the wounds were captured directly after insert removal and after 2, 4, 6, 8, and 10 h of observation on Leica light microscope (DMi1, Watzlar, Germany).

### 4.11. Molecular Dynamics Simulations

The MD simulations were performed using the GROMACS 2018.3 software on the computational cluster at the Department of Theoretical Chemistry and Physics of the University of Lorraine. The systems were prepared with the CHARMM-GUI software and visually inspected with VMD. The membrane model was composed of a lipid bilayer composed of 40% POPC, 30% POPEE, 16% POPCE, 4% POPCE, and 10% Cholesterol. 10 calcium ions were added to the system, by replacing two Na^+^ cations for one calcium ion in the NaCl solution. The system was initially minimized and equilibrated. Afterwards, 100 ns additional equilibration at constant temperature and constant pressure (NPT simulation) was applied to achieve the lack of surface tension across the simulated membrane. In order to induce transmembrane voltage and mimic the conditions of the EP experiments, we followed the protocols of the charge imbalance developed recently [73,74]: The box size was first extended in the *z*-axis to separate both water baths. Next, a short (10 ns) constant volume and constant temperature (NVT simulation) was performed to evaluate the surface tension on the vacuum-water interphase. This surface tension was applied in the subsequent constant temperature and constant surface tension (NPγT) simulation protocol, which was used to simulate electroporation and still allow as in experiment for a complete relaxation of the membrane surface tension. An ion imbalance of 8 electric charges was applied to induce a transmembrane potential large enough to induce the lipid bilayer electroporation. The simulation described was conducted for over 10 ns and the results were analyzed and visualized using VMD.

### 4.12. Statistical Analysis

The statistical analysis was performed using the GraphPad Prism 7 (La Jolla, CA, USA). Differences in cell viability were analyzed by two- way ANOVA or student *t*-test depending on the experiment. Results were expressed as mean ± standard deviation of the mean and with *p* < 0.05 being considered statistically significant.

## 5. Conclusions

This research confirms that calcium electroporation is a potent anti-PCa therapy in vitro. In the future it could potentially be used as an alternative, minimally-invasive focal therapy for prostate cancer. The hallmark of the CaEP is its safety and relatively easy applicability. In the nearest future, we can expect more data from clinical trials, and in case of promising results, extending the therapy to other cancers such prostate adenocarcinoma.

## References

[1] Neumann E., Rosenheck K. (1972). Permeability changes induced by electric impulses in vesicular membranes. J. Membr. Biol..

[2] Sale J.H., Hamilton W.A. (1968). Effects of high electric fields on micro-organisms III. Lysis of erythrocytes and protoplasts. Biochim. Biophys. Acta.

[3] Davalos R.V., Mir L.M., Rubinsky B. (2005). Tissue Ablation with Irreversible Electroporation. Ann. Biomed. Eng..

[4] Kalra N., Gupta P., Gorsi U., Bhujade H., Chaluvashetty S.B., Duseja A., Singh V., Dhiman R.K., Chawla Y.K., Khandelwal N. (2019). Irreversible Electroporation for Unresectable Hepatocellular Carcinoma: Initial Experience. Cardiovasc. Interv. Radiol..

[5] Ruarus A.H., Vroomen L.G.P.H., Geboers B., Van Veldhuisen E., Puijk R.S., Nieuwenhuizen S., Besselink M.G., Zonderhuis B.M., Kazemier G., De Gruijl T.D. (2020). Percutaneous Irreversible Electroporation in Locally Advanced and Recurrent Pancreatic Cancer (PANFIRE-2): A Multicenter, Prospective, Single-Arm, Phase II Study. Radiology.

[6] Trimmer C.K., Khosla A., Morgan M., Stephenson S.L., Ozayar A., Cadeddu J.A. (2015). Minimally Invasive Percutaneous Treatment of Small Renal Tumors with Irreversible Electroporation: A Single-Center Experience. J. Vasc. Interv. Radiol..

[7] Van Den Bos W., Scheltema M.J., Siriwardana A.R., Kalsbeek A.M.F., Thompson J., Ting F., Böhm M., Haynes A.-M., Shnier R., Delprado W. (2017). Focal irreversible electroporation as primary treatment for localized prostate cancer. BJU Int..

[8] Blazevski A., Scheltema M.J., Yuen B., Masand N., Nguyen T.V., Delprado W., Shnier R., Haynes A.-M., Cusick T., Thompson J. (2020). Oncological and Quality-of-life Outcomes Following Focal Irreversible Electroporation as Primary Treatment for Localised Prostate Cancer: A Biopsy-monitored Prospective Cohort. Eur. Urol. Oncol..

[9] Rosazza C., Meglic S.H., Zumbusch A., Rols M.-P., Miklavcic D. (2016). Gene Electrotransfer: A Mechanistic Perspective. Curr. Gene Ther..

[10] Daud A.I., DeConti R.C., Andrews S., Urbas P., Riker A.I., Sondak V.K., Munster P.N., Sullivan D.M., Ugen K.E., Messina J.L. (2008). Phase I Trial of Interleukin-12 Plasmid Electroporation in Patients With Metastatic Melanoma. J. Clin. Oncol..

[11] Mir L.M., Orlowski S., Belehradek J., Paoletti C. (1991). Electrochemotherapy potentiation of antitumour effect of bleomycin by local electric pulses. Eur. J. Cancer Clin. Oncol..

[12] Miklavčič D., Mali B., Kos B., Heller R., Sersa G. (2014). Electrochemotherapy: From the drawing board into medical practice. Biomed. Eng. Online.

[13] Chakrabarti R., E Wylie D., Schuster S.M. (1989). Transfer of monoclonal antibodies into mammalian cells by electroporation. J. Biol. Chem..

[14] Kinosita K., Tsong T.Y. (1978). Survival of sucrose-loaded erythrocytes in the circulation. Nat. Cell Biol..

[15] Cemazar M., Sersa G. (2019). Recent Advances in Electrochemotherapy. Bioelectricity.

[16] Kiełbik A., Szlasa W., Saczko J., Kulbacka J. (2020). Electroporation-Based Treatments in Urology. Cancers.

[17] Frandsen S.K., Gissel H., Hojman P., Tramm T., Eriksen J., Gehl J. (2012). Direct Therapeutic Applications of Calcium Electroporation to Effectively Induce Tumor Necrosis. Cancer Res..

[18] Falk H., Matthiessen L., Wooler G., Gehl J. (2017). Calcium electroporation for treatment of cutaneous metastases; a randomized double-blinded phase II study, comparing the effect of calcium electroporation with electrochemotherapy. Acta Oncol..

[19] Plaschke C.C., Gehl J., Johannesen H.H., Fischer B.M., Kjaer A., Lomholt A.F., Wessel I. (2019). Calcium electroporation for recurrent head and neck cancer: A clinical phase I study. Laryngoscope.

[20] Ágoston D., Baltás E., Ócsai H., Rátkai S., Lázár P.G., Korom I., Varga E., Nemeth I.B., Viharosné É.D.-R., Gehl J. (2020). Evaluation of Calcium Electroporation for the Treatment of Cutaneous Metastases: A Double Blinded Randomised Controlled Phase II Trial. Cancers.

[21] Rawla P. (2019). Epidemiology of Prostate Cancer. World J. Oncol..

[22] Zerlay J., Colombet M., Soerjomataram I., Mathers C., Parkin D.M., Piñeros M., Znaor A., Bray F. (2018). Estimating the global cancer incidence and mortality in 2018: GLOBOCAN sources and methods. Int. J. Cancer.

[23] Hamdy F.C., Donovan J.L., Lane J.A., Mason M., Metcalfe C., Holding P., Davis M., Peters T.J., Turner E.L., Martin R.M. (2016). 10-Year Outcomes after Monitoring, Surgery, or Radiotherapy for Localized Prostate Cancer. N. Engl. J. Med..

[24] Donaldson I.A., Alonzi R., Barratt D., Barret E., Berge V., Bott S., Bottomley D., Eggener S., Ehdaie B., Emberton M. (2015). Focal Therapy: Patients, Interventions, and Outcomes—A Report from a Consensus Meeting. Eur. Urol..

[25] Nassiri N., Chang E., Lieu P., Priester A.M., Margolis D.J.A., Huang J., Reiter R.E., Dorey F.J., Marks L.S., Natarajan S. (2018). Focal Therapy Eligibility Determined by Magnetic Resonance Imaging/Ultrasound Fusion Biopsy. J. Urol..

[26] Krimphove M.J., Cole A.P., Fletcher S.A., Harmouch S.S., Berg S., Lipsitz S.R., Sun M., Nabi J., Nguyen P.L., Hu J.C. (2018). Evaluation of the contribution of demographics, access to health care, treatment, and tumor characteristics to racial differences in survival of advanced prostate cancer. Prostate Cancer Prostatic Dis..

[27] Frandsen S.K., Vissing M., Gehl J. (2020). A Comprehensive Review of Calcium Electroporation—A Novel Cancer Treatment Modality. Cancers.

[28] Frandsen S.K., Gehl J. (2018). A Review on Differences in Effects on Normal and Malignant Cells and Tissues to Electroporation-Based Therapies: A Focus on Calcium Electroporation. Technol. Cancer Res. Treat..

[29] Hansen E.L., Sözer E.B., Romeo S., Frandsen S.K., Vernier P.T., Gehl J. (2015). Correction: Dose-Dependent ATP Depletion and Cancer Cell Death following Calcium Electroporation, Relative Effect of Calcium Concentration and Electric Field Strength. PLoS ONE.

[30] Frandsen S.K., Krüger M.B., Mangalanathan U.M., Tramm T., Mahmood F., Novak I., Gehl J. (2017). Normal and Malignant Cells Exhibit Differential Responses to Calcium Electroporation. Cancer Res..

[31] Szewczyk A., Gehl J., Daczewska M., Saczko J., Frandsen S.K., Kulbacka J. (2018). Calcium electroporation for treatment of sarcoma in preclinical studies. Oncotarget.

[32] Frandsen S.K., Gissel H., Hojman P., Eriksen J., Gehl J. (2014). Calcium electroporation in three cell lines: A comparison of bleomycin and calcium, calcium compounds, and pulsing conditions. Biochim. Biophys. Acta (BBA)-Gen. Subj..

[33] Hoejholt K.L., Mužić T., Jensen S.D., Dalgaard L.T., Bilgin M., Nylandsted J., Heimburg T., Frandsen S.K., Gehl J. (2019). Calcium electroporation and electrochemotherapy for cancer treatment: Importance of cell membrane composition investigated by lipidomics, calorimetry and in vitro efficacy. Sci. Rep..

[34] Walsh P.C. (2012). Re: The Natural History of Metastatic Progression in Men with Prostate-Specific Antigen Recurrence After Radical Prostatectomy: Long-Term Follow-up. J. Urol..

[35] Blazevski A., Scheltema M.J., Amin A., Thompson J., Lawrentschuk N., Stricker P.D. (2019). Irreversible electroporation (IRE): A narrative review of the development of IRE from the laboratory to a prostate cancer treatment. BJU Int..

[36] Palmer T.D., Ashby W.J., Lewis J.D., Zijlstra A. (2011). Targeting tumor cell motility to prevent metastasis. Adv. Drug Deliv. Rev..

[37] Brock R.M., Beitel-White N., Davalos R.V., Allen I.C. (2020). Starting a Fire without Flame: The Induction of Cell Death and Inflammation in Electroporation-Based Tumor Ablation Strategies. Front. Oncol..

[38] Bioquest® A. Fluo-8® Calcium Reagents and Screen Quest TM Fluo-8 NW Calcium Assay Kits. https://docs.aatbio.com/products/protocol/A3300d1.pdf.

[39] Rudel T. (1999). Caspase inhibitors in prevention of apoptosis. Herz.

[40] Li J.S., Yuan J. (2008). Caspases in apoptosis and beyond. Oncogene.

[41] Clapham D.E. (2007). Calcium Signaling. Cell.

[42] Furuya Y., Lundmo P., Short A.D., Gill D.L., Isaacs J.T. (1994). The role of calcium, pH, and cell proliferation in the programmed (apoptotic) death of androgen-independent prostatic cancer cells induced by thapsigargin. Cancer Res..

[43] Novickij V., Čėsna R., Perminaitė E., Zinkevičienė A., Characiejus D., Novickij J., Šatkauskas S., Ruzgys P., Girkontaitė I. (2019). Antitumor Response and Immunomodulatory Effects of Sub-Microsecond Irreversible Electroporation and Its Combination with Calcium Electroporation. Cancers.

[44] Staresinic B., Jesenko T., Kamensek U., Frandsen S.K., Sersa G., Gehl J., Cemazar M. (2018). Effect of calcium electroporation on tumour vasculature. Sci. Rep..

[45] Szewczyk A., Saczko J., Kulbacka J. (2020). Apoptosis as the main type of cell death induced by calcium electroporation in rhabdomyosarcoma cells. Bioelectrochemistry.

[46] Zielichowska A., Daczewska M., Saczko J., Michel O., Kulbacka J. (2016). Applications of calcium electroporation to effective apoptosis induction in fibrosarcoma cells and stimulation of normal muscle cells. Bioelectrochemistry.

[47] Verkhratsky A. (2007). Calcium Signalling and Disease. Sub-Cell. Biochem..

[48] Zhivotovsky B., Orrenius S. (2011). Calcium and cell death mechanisms: A perspective from the cell death community. Cell Calcium.

[49] Cerella C., Diederich M., Ghibelli L. (2010). The Dual Role of Calcium as Messenger and Stressor in Cell Damage, Death, and Survival. Int. J. Cell Biol..

[50] Gibot L., Montigny A., Baaziz H., Fourquaux I., Audebert M., Rols M.-P. (2020). Calcium Delivery by Electroporation Induces In Vitro Cell Death through Mitochondrial Dysfunction without DNA Damages. Cancers.

[51] Romeo S., Sannino A., Scarfì M.R., Vernier P.T., Cadossi R., Gehl J., Zeni O. (2018). ESOPE-Equivalent Pulsing Protocols for Calcium Electroporation: An In Vitro Optimization Study on 2 Cancer Cell Models. Technol. Cancer Res. Treat..

[52] Falk H., Forde P.F., Bay M.L., Mangalanathan U.M., Hojman P., Soden D.M., Gehl J. (2017). Calcium electroporation induces tumor eradication, long-lasting immunity and cytokine responses in the CT26 colon cancer mouse model. OncoImmunology.

[53] Wolf A., Wennemuth G. (2013). Ca^2+^ clearance mechanisms in cancer cell lines and stromal cells of the prostate. Prostate.

[54] Flourakis M., Prevarskaya N. (2009). Insights into Ca^2+^ homeostasis of advanced prostate cancer cells. Biochim. Biophys. Acta (BBA)-Bioenergy.

[55] Frandsen S.K., Gibot L., Madi M., Gehl J., Rols M.-P. (2015). Calcium Electroporation: Evidence for Differential Effects in Normal and Malignant Cell Lines, Evaluated in a 3D Spheroid Model. PLoS ONE.

[56] Prevarskaya N., Skryma R., Shuba Y. (2004). Ca^2+^ homeostasis in apoptotic resistance of prostate cancer cells. Biochem. Biophys. Res. Commun..

[57] Cui C., Merritt R., Fu L., Pan Z. (2017). Targeting calcium signaling in cancer therapy. Acta Pharm. Sin. B.

[58] Olson M.F., Sahai E. (2009). The actin cytoskeleton in cancer cell motility. Clin. Exp. Metastasis.

[59] Graybill P.M., Davalos R.V. (2020). Cytoskeletal Disruption after Electroporation and Its Significance to Pulsed Electric Field Therapies. Cancers.

[60] Roy S.S., Hajnóczky G. (2008). Calcium, mitochondria and apoptosis studied by fluorescence measurements. Methods.

[61] Hanna H., Denzi A., Liberti M., André F.M., Mir L.M. (2017). Electropermeabilization of Inner and Outer Cell Membranes with Microsecond Pulsed Electric Fields: Quantitative Study with Calcium Ions. Sci. Rep..

[62] Kim H.B., Lee S., Chung J.H., Kim S.N., Sung C.K., Baik K.Y. (2020). Effects of Actin Cytoskeleton Disruption on Electroporation In Vitro. Appl. Biochem. Biotechnol..

[63] Gimsa J., Wachner D. (2001). Analytical Description of the Transmembrane Voltage Induced on Arbitrarily Oriented Ellipsoidal and Cylindrical Cells. Biophys. J..

[64] Vernier P.T., Ziegler M.J., Dimova R. (2009). Calcium Binding and Head Group Dipole Angle in Phosphatidylserine−Phosphatidylcholine Bilayers. Langmuir.

[65] Levine Z.A., Ziegler M.J., Vernier P.T. (2010). Life Cycle of an Electropore: A Molecular Dynamics Investigation of the Electroporation of Heterogeneous Lipid Bilayers (PC:PS) In the Presence of Calcium Ions. Biophys. J..

[66] Navickaite D., Ruzgys P., Novickij V., Jakutaviciute M., Maciulevičius M., Sinceviciute R., Šatkauskas S. (2020). Extracellular-Ca^2+^-Induced Decrease in Small Molecule Electrotransfer Efficiency: Comparison between Microsecond and Nanosecond Electric Pulses. Pharmaceutics.

[67] Falk H., Lambaa S., Johannesen H.H., Wooler G., Venzo A., Gehl J. (2017). Electrochemotherapy and calcium electroporation inducing a systemic immune response with local and distant remission of tumors in a patient with malignant melanoma–A case report. Acta Oncol..

[68] Rudno-Rudzińska J., Kielan W., Guziński M., Płochocki M., Kulbacka J. (2020). The First Study of Irreversible Electroporation with Calcium Ions and Chemotherapy in Patients with Locally Advanced Pancreatic Adenocarcinoma. Appl. Sci..

[69] Klein N., Gunther E., Zapf S., El-Idrissi R., Atta J., Stehling M. (2017). Prostate cancer infiltrating the bladder sphincter successfully treated with Electrochemotherapy: A case report. Clin. Case Rep..

[70] Nielsen K., Scheffer H.J., Vieveen J.M., Van Tilborg A.A.J.M., Meijer S., Van Kuijk C., Van Den Tol M.P., Meijerink M.R., Bouwman R.A. (2014). Anaesthetic management during open and percutaneous irreversible electroporation. Br. J. Anaesth..

[71] Napotnik T.B., Miklavčič D. (2018). In vitro electroporation detection methods—An overview. Bioelectrochemistry.

[72] Schindelin J., Arganda–Carrera I., Frise E., Verena K., Mark L., Tobias P., Stephan P., Curtis R., Stephan S., Benjamin S. (2009). Fiji-an open platform for biological image analysis. Nat. Methods.

[73] Casciola M., Tarek M. (2016). A molecular insight into the electro-transfer of small molecules through electropores driven by electric fields. Biochim. Biophys. Acta (BBA)-Biomembr..

[74] Casciola M., Kasimova M.A., Rems L., Zullino S., Apollonio F., Tarek M. (2016). Properties of lipid electropores I: Molecular dynamics simulations of stabilized pores by constant charge imbalance. Bioelectrochemistry.

